# History of Technological Advancements towards MR-Linac: The Future of Image-Guided Radiotherapy

**DOI:** 10.3390/jcm11164730

**Published:** 2022-08-12

**Authors:** Nikhil Rammohan, James W. Randall, Poonam Yadav

**Affiliations:** Department of Radiation Oncology, Northwestern Medicine, Chicago, IL 60611, USA

**Keywords:** magnetic resonance, linear accelerator, MRgRT, radiotherapy, image-guided radiotherapy, IGRT, adaptive therapy

## Abstract

Image-guided radiotherapy (IGRT) enables optimal tumor targeting and sparing of organs-at-risk, which ultimately results in improved outcomes for patients. Magnetic resonance imaging (MRI) revolutionized diagnostic imaging with its superior soft tissue contrast, high spatiotemporal resolution, and freedom from ionizing radiation exposure. Over the past few years there has been burgeoning interest in MR-guided radiotherapy (MRgRT) to overcome current challenges in X-ray-based IGRT, including but not limited to, suboptimal soft tissue contrast, lack of efficient daily adaptation, and incremental exposure to ionizing radiation. In this review, we present an overview of the technologic advancements in IGRT that led to MRI-linear accelerator (MRL) integration. Our report is organized in three parts: (1) a historical timeline tracing the origins of radiotherapy and evolution of IGRT, (2) currently available MRL technology, and (3) future directions and aspirations for MRL applications.

## 1. Introduction

We live in an exciting era of radiotherapy due to a myriad of technological innovations, creating superior dose to target and minimum dose to organs at risk (OAR). We have seen numerous advancements in image-guided radiotherapy (IGRT) [1,2], adaptive treatment planning [3,4,5], and automated segmentation [5], all of which have led to more efficient treatment delivery and better outcomes for cancer patients. Along with proton beam therapy [6], the integration of MRI with a traditional linear accelerator (Linac) has been at the forefront of these advances. In this review, we present an overview of the technologic advancements that led to the development of MRL, as well as the current and future applications of the technology towards more accurate and precise radiotherapy with a focus on online adaptive therapy.

## 2. History of Technologic Advancements in IGRT

### 2.1. The Origins of Radiotherapy—Dose and Fractionation

Wilhelm Roentgen’s discovery of X-ray photons in 1895 enabled the visualization of internal anatomical structures within a live, intact patient for the first time [7,8]. The discovery transformed the capabilities of medicine, as opaque subjects became transparent for diagnosis, treatment, and research. It was observed that patients with malignancies who were exposed for prolonged periods to the photon-rays would have regression of disease [7,9,10]. Termed “Roentgen Therapy”, these crude treatments were the beginning of a new field of medicine, Therapeutic Radiology, later termed Radiation Oncology [11,12].

With improvement in the understanding of cellular biology, it was ultimately determined that the mechanism behind cell killing in radiotherapy involved structures within the nucleus [13,14,15]. The underlying mechanism was eventually shown to be induction of DNA damage from radiation, which could render the cells no longer reproductively viable. Whether treating malignant or benign processes, the therapeutic effect of radiation lies in disrupting DNA-structure and prohibiting cellular replication [13,14,15].

In the early days of radiotherapy, much like today, significant effort was focused on enhancing the therapeutic index—maximizing disease response and minimizing unwanted toxicity. One of the first practitioners of radiotherapy, Leopold Freund, observed that using small daily radiation doses caused hairy nevi to regress [16]. Dr. Freund utilized this prolonged approach because the X-ray tubes at the time were only capable of a low dose rate, and multiple successive treatments were required to achieve the desired response. As rapidly developing X-ray tube technology allowed for higher dose rates, physicians started to observe that treating patients with higher doses in fewer treatments resulted in severe toxicity. Therefore, it appeared that Dr. Freund had accidentally discovered the therapeutic value of fractionation—breaking up the prescribed dose into smaller treatments to arrest the growth of malignant tissue and allow for the repair of slow-dividing surrounding healthy tissue [13,14,15].

The benefits of fractionation are tissue specific—for example, breast and prostate tissue respond more favorably to higher doses over fewer fractions, while epithelial tissues of the head and neck respond more favorably to lower doses over a higher number of fractions [17,18,19,20]. This was also compounded by the fact that accuracy in location of tumor was limited. By fractionating treatments, there was a high probability that the target remained in the treatment field.

Modern radiotherapy is trending from conventional fractionation to hypofractionation for most cancer subsites. For example, conventional breast radiotherapy has consisted of 25 to 30 treatments to a dose of 50–60 Gy. However, modern hypofractionated regimens typically consist of 15 to 20 treatments reaching a dose of about 40–45 Gy and have become widely utilized [18,21,22]. These hypofractionated regimens have several advantages, including reduced acute toxicity, better treatment tolerance, and reduced treatment time [18,21,22]. In fact, there is increasing acceptance of ultrahypofractionation—usually consisting of five or fewer treatments with relatively large doses delivered daily [23]. Due to the increased dose per fraction, these treatments require higher anatomical confidence and techniques to minimize set-up variation and maximize confidence in target location. Thanks to modern treatment machines, which combine on-board imaging with beam delivery, improved accuracy in target localization is possible. Hypo- and ultrahypofractionation would not be as accessible without modern IGRT, which has proved paramount for modern treatment.

### 2.2. The Rise of Image Guidance in Radiotherapy

The desire to see the clinical target during treatment led to IGRT innovation, which enables optimal targeting of the desired tissues and limits the dose to OARs. In the early days of clinical radiotherapy, fluoroscopic imaging of patients was utilized for preemptive planning of radiotherapy [24]. This allowed the treatment team to visualize underlying bony anatomy and get a rough idea of soft tissue distribution that was specific to each patient. Moving from a “point and shoot” approach, this change brought the first steps toward the standardization of radiotherapy. Bony landmarks were used to define borders, and lead blocks were utilized to block radiation from direct contact with underlying critical structures such as the heart or kidneys [11,24].

The introduction of computed tomography (CT), which was developed in 1972 [25], revolutionized X-ray imaging, and patient treatment was significantly advanced in the early 1990s with the production of an algorithm that could reconstruct a three-dimensional (3D) image [26,27]. Patients would now undergo a CT scan in their treatment position, with planning of radiotherapy performed in 3D. This leads to better understanding of dose distribution, avoids OARs, and provides more conformal coverage of targets [28].

**Figure 1:**
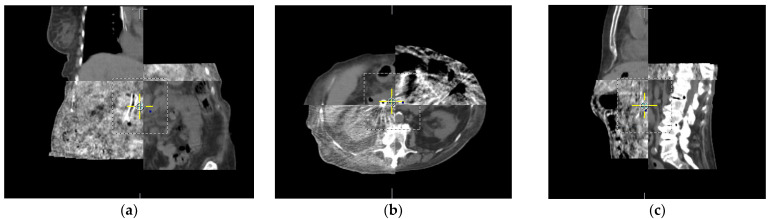
Registration of cone beam CT obtained in treatment position on treatment machine to planning CT scan for abdominal RT in three dimensions: (**a**) coronal, (**b**) axial, and (**c**) sagittal slices each illustrate the registration between the CT scans used on the treatment machine to ensure alignment.

An extension of CT technology for cone-beam CT (CBCT), developed by David Jaffray [29,30], was utilized on the treatment machine by taking advantage of onboard imagers that provided pre-treatment CT-like images to match the planned treatment volume by image fusion, as shown in Figure 1. This provided the initial impetus for IGRT.

Patient position changes can occur due to breathing, pain, involuntary muscular changes, and autonomic organ motion, notably in the gastrointestinal tract and cardium. Positional discrepancies can range from a few millimeters to several centimeters. To account for these changes, IGRT must look beyond X-ray technologies alone. David Jaffray elaborates that IGRT is also crucial to limit dose to OARs, more dose delivery to target tumor, increasing dose per fraction, dose distribution based on target heterogeneity, and detection of treatment changes in tissue [2]. Developing IGRT systems that can tackle these multiple paradigms of treatment simultaneously is a lofty goal but has potential to improve treatment outcomes.

### 2.3. Technical Evolution of Treatment Conformality

The two-dimensional (2D) RT era largely involved rudimentary calculations of dose, because there could be only a limited number of simple square or rectangular beams produced by physical blocks and wedges [11,24]. The advent of CT led to the three-dimensional era where treatment planning became more complex. The first 3D treatment planning systems (TPSs) were developed in the early 1990s and were able to utilize patient anatomy and electron density from the CT images to guide beam geometry according to the prescribed treatment plan [31]. The TPS was further able to generate dose-volume histograms, which allowed for optimal assessment of dose to target versus dose to OARs [31]. These advances, along with IGRT as described above, allowed for generation of much more conformal treatment plans than was possible in the 2D era. These TPSs allowed “forward planning”, where beam parameters were manually specified to achieve the desired dose distribution.

**Figure 2:**
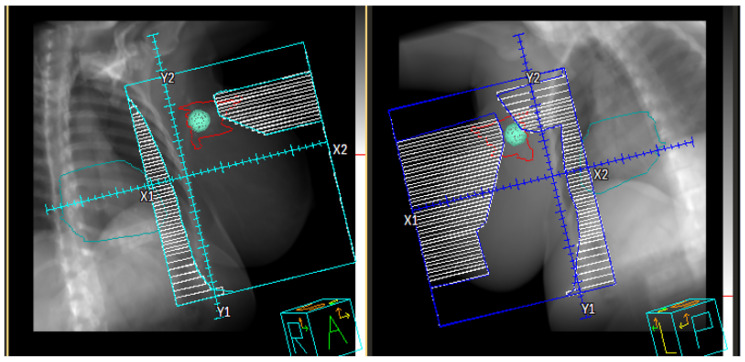
Step and Shoot IMRT with tangential approach used for treating breast cancer. Note the interdigitation of the MLCs that are used to modulate dose received with each treatment segment or configuration of leaves.

**Figure 3:**
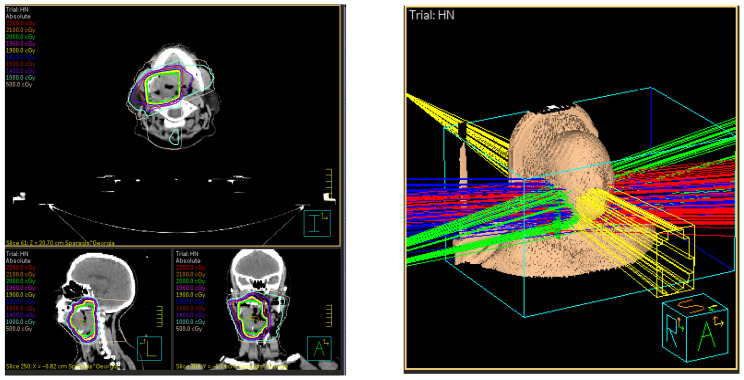
Inverse Planning IMRT used to create a more complex treatment plan for a head and neck case. Note the highly conformal dose distribution (**left**) panel and the multiple beams (**right**) panel, each with a unique MLC configuration, as can be noted on yellow beam.

**Figure 4:**
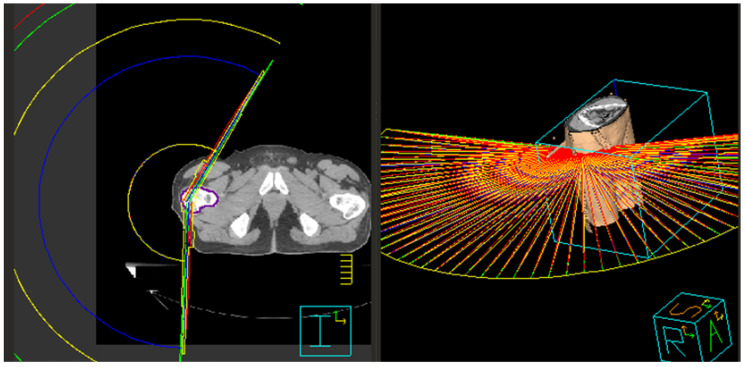
Volumetric modulated arc therapy treatment technique illustrated in (**left**) two dimensions and (**right**) three dimensions. Note the sweeping, continuous arc motion around the patient.

In the late 1990s, there was a concerted approach to further refine treatment planning that led to the development of intensity-modulated RT (IMRT), where the intensity of the radiation beam was modulated according to the shape and volume of the target [32,33,34]. The earliest versions of IMRT were static, termed “step-and-shoot” (S&S) and “sliding window” (SW), where the dose was delivered from a discrete number of beam angles (Figure 2) [35]. Both techniques allow for “painting” a continuous pattern of radiation density [35]. Often, IMRT required “inverse planning”, where an optimal beam arrangement was computed by an algorithm that accounts for planning target volumes (PTVs) and OAR volumes as inputs (Figure 3). While the final treatment plan was highly conformal, a major disadvantage of early IMRT (S&S, SW) was the prolonged treatment time because intensity modulation required higher machine output to deliver a dose distribution respecting the prescription boundaries of the targets and OARs [32,33]. A further advancement of IMRT was volumetric modulated arc therapy (VMAT), where beams were delivered with continuous rotation of the Linac gantry and multileaf collimators (MLCs) with variable dose rate (Figure 4) [36,37]. This enabled far shorter treatment times and is currently the preferred form of highly conformal RT.

### 2.4. Adaptive Treatment Planning

Adaptive treatment planning refers to using on-board imagers to modify treatments on a daily basis. Adapting can account for day-to-day variations in patient characteristics; as such, it represents the confluence of IGRT and conformal treatment planning to achieve the most accurate dose and highest therapeutic index. Improvement in volumetric imaging and availability of auto-segmentation allows daily dose to be computed, thereby treatment planning decisions can be made based on dosimetric changes as opposed to geometric changes alone [38,39,40]. Further, emerging studies demonstrate that functional changes within the tumor (e.g., change in PET avidity, necrosis within the tumor) can be incorporated during adaptation [41,42].

Adaptive planning can be further classified into online and offline adaptation [43,44]. The former refers to changes in the treatment plan that are made while the patient remains on the treatment table in the treatment position, while the latter refers to changes made to the treatment plan that occur between fractions. Each has advantages; for instance, online adaptation can correct for any changes in anatomy before delivering radiation. Offline adaptation prevents the need for the patient to remain immobilized in the treatment position while addressing anatomical or treatment response changes.

### 2.5. A Brief History of Magnetic Resonance Imaging

The foundation for MRI as a potential imaging modality can be largely attributed to the work of two pioneering scientists: Raymond Damadian and Paul Lauterbur. In 1971, Raymond Damadian demonstrated that tumors could potentially be detected by their vastly different nuclear magnetic resonance (NMR) relaxation times as compared to healthy tissue [45]. While he did not provide a technique for how an image of a patient or portion of a patient could be obtained, it was perhaps the first indication that the principles of NMR could be utilized in medical imaging.

In his seminal Nature paper in 1973, Paul Lauterbur suggested “a new class of image generated, by taking advantage of induced local interactions” [46]. Up until that time, the prevalent theory was that the generation of an image required the object to interact with matter or radiation with a wavelength equal or less than the smallest feature to be delineated. Lauterbur’s idea was to use the principles of NMR; his solution was to place the object in a secondary magnetic field such that the object’s interaction with the primary magnetic field would be restricted to a particular region, thereby making the image measurement independent of wavelength. He described this new technique as “NMR zeugmatography” [46] for general purpose imaging not specific to medicine. Peter Mansfield extended Lauterbur’s initial findings by incorporating frequency and phase encoding via spatial magnetic field gradients. He subsequently developed the mathematical analysis of the RF signals from MRI, which eventually led to image development [47].

While a detailed explanation of NMR relaxation theory can be found in a number of excellent articles [48,49,50,51], a brief overview is presented here. The signal-to-noise ratios (SNR) in MRI depend on the density of water protons (the human body is roughly 60% water) present in the region of interest and the degree of polarization of the nuclear spin states. When placed in a magnetic field, some protons will orient in the direction of the magnetic field. Upon application of an external radiofrequency (RF) pulse, at the resonant frequency, the proton spins are perturbed out of alignment with the magnetic field and process at a characteristic frequency (known as Larmor frequency) related to the strength of the magnetic field. Once the pulse is removed, the protons return (“relax”) back to their ground state. Relaxation is measured in two directions: longitudinal and transverse. Longitudinal or spin-lattice relaxation is defined by the time constant T1 and occurs in the direction of the main magnetic field. Transverse or spin–spin relaxation corresponds to vector dephasing in the plane perpendicular to the main magnetic field and is characterized by T2. T2 is always equal to or shorter than T1. Inhomogeneity in the static magnetic field and spin–spin relaxation has an effect on the transverse magnetization, and T2* is the time constant that takes these into account. Overall, the time constants always adhere to the following relation: T2* < T2 < T1. Signals received from spin vectors are used to produce images by superimposing magnetic gradients that define the spatial location of the signal. Tissue types vary in their relaxation properties, and thus MRI is used to reconstruct images to evaluate anatomical, perfusion, and flow-related abnormalities [48,49,50,51].

MRI offers several advantages over the other medical imaging techniques. Compared to X-ray radiography, CT, positron emission tomography (PET), and single-photon emission computed tomography (SPECT), MRI provides exceptional soft tissue contrast, has no limits to depth penetration, does not utilize nor produce ionizing radiation, and enables imaging in three dimensions [48,51,52,53,54]. Compared to ultrasound or optical modalities, it has far superior spatial resolution (as low as 50 μm depending on field strength of the magnet) [48,55,56].

## 3. Current MRgRT Systems and Applications

### 3.1. Advancing from CT to MRI-Guided Radiotherapy

As demonstrated, image guidance has progressed tremendously in the short existence of radiotherapy. With each jump in technology, treatment outcomes have improved in parallel. A well-known demonstration is the reduction of salivary toxicity in the treatment of oropharyngeal carcinoma with the adoption of IMRT planning [57]. Similar patterns of toxicity reduction with use of IMRT have also been observed in anal cancer [58] and post-operative endometrial cancer [59].

The same is true for the field of radiological imaging; better visualization of malignancy can lead to improved oncologic care. Recent evidence is provided by the use of MRI in the treatment of prostatic malignancies. Multiparametric MRI uses multiple sequences that each provide unique information regarding prostatic malignancy, which has greatly advanced the diagnostic capability and staging of prostate cancer [60]. Such informative imaging has been used in the delivery of radiotherapy to improve the targeting of gross disease within the prostate gland, which has been shown to increase biochemical disease-free survival in a randomized trial [61].

The current standard of image-guided radiotherapy consists of on-board kilovoltage X-ray imaging, cone beam CT imaging, megavoltage CT, and CT on rails that are used to ensure accurate treatment position [28]. After obtaining on-board images, small adjustments of the table positioning can be made on a daily basis to properly align the target or avoidance structures. The objective is to recreate the initial setup for accurate administration of the predicted radiotherapy plan. The limited adaptation provided by current systems gives little opportunity for plan adjustment per fraction. Furthermore, CT-guided systems provide relatively poor soft tissue imaging for off-table or on-table planning compared to on-board MRI, as rated by planning physicians [62].

The goal of image-guided radiotherapy should be a seamless incorporation of diagnostic radiology in traditional oncology to improve delivery of radiotherapy and allow for further degrees of freedom in daily treatments, adaptation, and better visualization. There are now multiple examples of therapeutic machines that have incorporated MRI on-board, with investigative trials underway [63,64]. These machines provide numerous benefits over the current, CT-based standard of care but also come with limitations that must be considered. Application of these machines in the clinic requires multidisciplinary coordination and holds the key to taking the next step in patient care during radiotherapy.

### 3.2. Current MR-Guided Systems

There are two widely adopted systems that have incorporated MRI into radiotherapy machines: the MRIdian from ViewRay (Oakwood, OH, USA) and the Unity from Elekta (Stockholm, Sweden).

#### 3.2.1. The ViewRay MRIdian

The components and setup of the ViewRay MRIdian have also been described in significant detail elsewhere [65] and are only briefly summarized here. The MRIdian principally consists of three components: (1) MRI system, (2) a RT delivery system, and (3) a TPS.

The MRIdian contains a “double donut”, horizontal, 0.35-T MRI, which includes a 50-cm spherical imaging field of view. The gradient coil, self-shielded against thrust and torque, has an inner diameter of 80 cm. The gradient field strength is 18 mT/m with a maximum slew rate of 200 T/m/s on each axis. The transmit coil is capable of covering the whole body and contains an integrated RF shield designed to span the magnet gap of 28 cm while remaining uniformly attenuating to prevent beam heterogeneities. The two receiving coils are the torso and head and neck coils with six- and five-element phased arrays, respectively, in each half. Both coils are made with flexible foam for ease of the patient.

**Figure 5:**
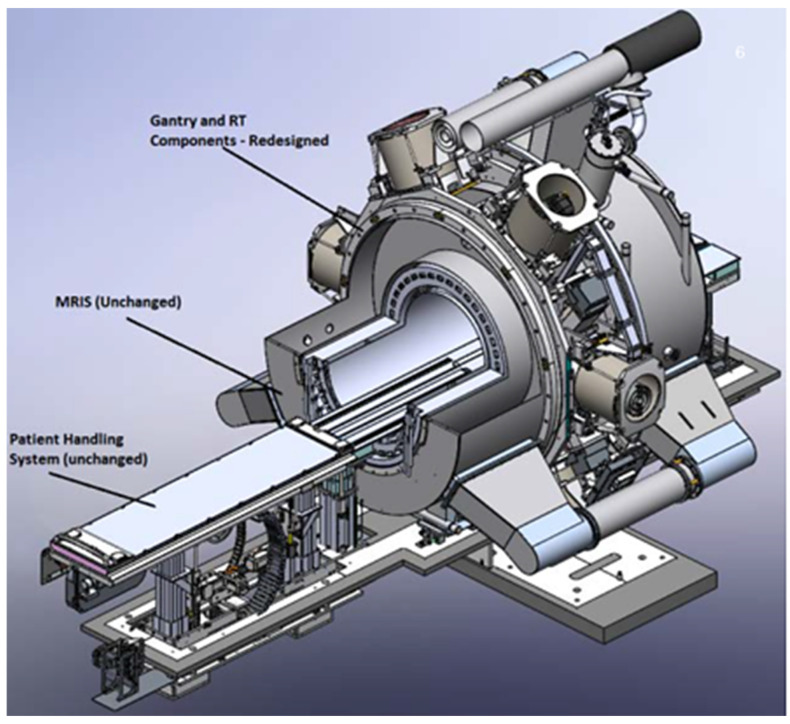
ViewRay MRIdian MRL model that replaced the initial tri-cobalt design in 2017.

In its initial iteration, the MRIdian contained a ring gantry housing three separate cobalt-60 sources that was capable of providing a dose rate of 550 cGy/min. A later update to the system included the integration of Linac, which can generate a 6 MV flattening filter-free photon beam at the nominal dose rate of 600 MU per minutes at 90 cm isocenter. Figure 5 shows the updated setup of ViewRay MRIdian. The Linac and its subcomponents are hidden from the MRI using five ferromagnetic steel concentric cylinders with three additional metal shields on the electron gun. MRI is shielded by RF-absorbing carbon fiber and RF-reflecting copper material. Integrated MRI enabled intrafraction tracking via rapid acquisition of planar images, while radiation beams could be gated by the presence of soft tissue of interest in the prescribed physical boundary.

The Viewray MRIdian TPS is capable of planning conformal RT, IMRT, and on-table adaptive RT with Monte Carlo-based dose computation. TPS is an integral part of the treatment delivery system, which help in performing on-table adaptation.

#### 3.2.2. The Elekta Unity

A detailed description of the Elekta Unity is published in multiple excellent articles [66,67] and is summarized herein. Briefly, the system is composed of a 7 MV Linac mounted circumferentially around a modified 1.5 T MRI system.

A modified 1.5 T Philips Achieva MRI system (Best, The Netherlands) is integrated with the Linac, but is capable of independent operation. In fact, all Achieva hardware such as cryocooler, cooling cabinet, gradient amplifier, RF coils, and RF amplifiers, as well as software scan protocols, are standard in the Unity; only a modified gradient coil and magnet were required to operate in conjunction with the Linac. In terms of the magnet, a custom 1.5 T magnet was built (Magnex, Oxford, UK). It contains a 15 cm central gap and allows for a maximal radiation field size of 24 cm in the craniocaudal direction at the isocenter. The magnet has active shielding, with two shield coils with opposing polarity, which generate a toroidal low-field (<10-4-T) zone where the most sensitive Linac components are housed. The gradient coil was custom built by Futura (Heerhugowaard, The Netherlands).

The Linac is positioned laterally to the MRI, with a source-isocenter distance of 150 cm. The Linac is a 7 MV Elekta (Crawley, UK) standing waveguide capable of 350 cGy/min dose rate at 100 cm. Steel components within the original freestanding Linac are replaced by non-ferromagnetic ones. The Linac is mounted on a custom wooden frame (as opposed to a steel gantry). The Linac and the MRI system are magnetically decoupled, allowing the Linac to operate independently.

The Elekta Unity is capable of planning IMRT and 3D conformal RT only. It contains a Monaco TPS capable of two types of adaptation—adapt-to-position (ATP) or adapt-to-shape (ATS). The ATP workflow adapts the plan according to patient position during treatment MRI compared to pre-treatment simulation CT; in this case, re-optimization is performed on the original contours. The ATS workflow optimizes the plan based on patient anatomy; in this situation, patient contours can be modified per physician discretion. The re-optimization time for ATP ranges from 18 to 376 s, and the ATS workflow ranges from 17 to 485 s, depending on case complexity [67].

#### 3.2.3. Comparison between ViewRay MRIdian and Elekta Unity

As detailed above, there are several differences between the two MRI-Linac systems. First, ViewRay MRIdian has a lower field-strength magnet (0.35 T vs. 1.5 T), which results in lower signal-to-noise ratio (30 dB vs. 120 dB respectively) but also lower electron return effect [68,69]. In terms of the Linac, MRIdian originally contained Co-60 sources, but newer versions contain a Linac capable of producing 6 MV FFF photon beams; however, the MRIdian operates at a higher dose rate (550–600 cGy/min compared to 350 cGy/min on the Unity). There are also differences in the range and speed of the MLCs: for MRIdian, the MLC range is 24.1 cm head-to-toe and 27.4 cm across the body, versus 22 cm and 57.4 cm, respectively for Unity. In terms of TPS, both are capable of adaptation as detailed above, but MRIdian is capable of real-time tracking. Both machines are limited to step-and-shoot IMRT, as arc therapies cause significant magnetic field perturbation from moving metallic components of the gantry and associated MLCs [63,70]. However, recent evidence has shown that the impact on image quality is often negligible and arc therapy could become feasible [70]. Despite the options, all machines function with the same underlying principle of centering MRI on the workflow of radiotherapy with the objective of the improved delivery of radiotherapy.

### 3.3. Other MRI-Linacs in Development

At this time, there are two additional MR-linear accelerators under investigation by separate teams that are not implemented clinically. The Aurora RT System from MagnetTx Oncology Solutions (Edmonton, AB, Canada) was one of the earlier machines to be investigated, first described in 2009 [71]. It utilizes a 0.5 T magnet with a 6 MV linear accelerator [64,71]. Finally, there is a prototype machine being developed in Sydney, Australia that uses a 1.0 T magnet with an open configuration and a fixed gantry [72]. This static approach is limited in that the patient must be manipulated to change the beam approach.

### 3.4. Advantages of MR-Guided Radiotherapy

As mentioned prior, many theoretical benefits of MRgRT exist. Ultimately, the objective is to take advantage of such benefits to increase the therapeutic index, improving radiation outcomes while minimizing toxicity.

**Figure 6:**
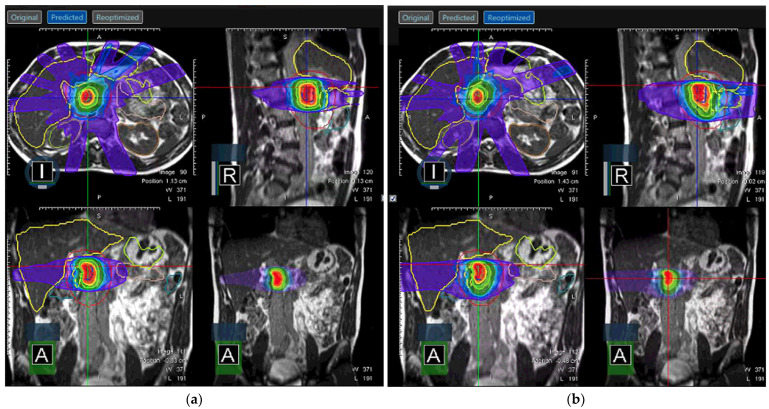
Adapted radiotherapy plan on ViewRay MRIdian for abdominal target. (**a**) The predicted plan was found to have inadequate target coverage and/or OAR sparing (blue color) and (**b**) was adapted to produce the reoptimized plan. Note the change in dose to stomach on the axial slice.

Soft tissue delineation is greatly improved in MRI when compared to CT and can help avoid OARs that would otherwise be crowded by organs of similar electron density on CT. Improved visualization of targets and organs at risk is then combined with the potential for treatment adaptation based on real-time imaging. Altering the initial radiotherapy plan to better fit patient anatomy is especially critical in a body with moving structures, such as those of the abdomen, where toxicity is a significant risk and organs lie in close adjacency (see Figure 6). Multiple studies have shown dosimetric benefit of increased target coverage and reduced dose to normal structures by utilization of adaptative radiotherapy afforded by MRgRT [73,74] with translation to good clinical outcomes [75,76].

Motion management is always considered in radiotherapy, with sources being either interfractional or intrafractional. MRgRT, a feature unique to the ViewRay MRIdian, allows for real-time MRI of structures, even though limited to the sagittal plane. By setting boundaries that trigger beam activation or cessation, precise target gating administers the dose only when the target falls within the predefined region [77]. The real time imaging and gating technique provide confidence in delivering high dose to the tumor and less normal tissue exposure to radiation, and theoretically reduced toxicity [78,79]. Changes in the positioning of internal organs between fractions can also be assessed with MRgRT and trigger adaptation of the predicted plan (plan with new MR image and edited structures but original plan dose). Similar dosimetric benefits have been observed in multiple sites using adaptation to account for interfractional motion [80,81,82].

A potential benefit of daily MRI of malignancies that are undergoing treatment lies in a limitation in the current standard of care. During the course of traditional radiotherapy, there is no assessment of treatment response; the same radiotherapy plan is traditionally applied from start to finish unless there is significant anatomic change. With a diversity of MR sequences, physiologic changes in the tissue can be exploited across the course of therapy and may serve as an imaging biomarker of response or persistence. Changes in MR during the course of radiotherapy have been investigated in a multitude of studies [83]. If clinically validated, these biomarkers could provide recommendations for dose escalation or de-escalation, or treatment prolongation or cessation. Similar to the aforementioned local boosting of gross disease in prostate cancer [61], areas of known higher cell content or hypoxia could be localized and targeted with more advanced MRI sequences. This technique was proven feasible by looking for changes on apparent diffusion coefficient mapping that was later correlated to the pathologic findings of resected tumors [84]. Finally, MRI is non-ionizing, as opposed to the series of verification kilovoltage images, CBCT’s, MVCT, or replanning CT’s required in traditional radiotherapy. MRLs can reduce non-therapeutic radiation exposure, which has large implications in sensitive populations (e.g., pediatrics).

In principle, each advantage can provide incremental benefit for the patient. While smaller trials have shown multiple instances of reduced toxicity with equivalent or improved oncologic outcomes, the feasibility of treatment with these machines faces multiple hurdles.

### 3.5. Limitations of MR-Guided Radiotherapy

Wider clinical use of MRLs has revealed limitations in the practical use of the machines, the dosimetry involved, and inherent flaws of MR-only procedures. The practical limitations of MRgRT, such as bore size and option for coplanar treatments, must be considered. Initial simulation on either an MR or CT planning scan must utilize the necessary MR compatible immobilizing equipment and RF coils, while still being able to fit into the limited diameter of the MRI bore. Similarly, RF coils must be placed in a position that does not alter the patient’s anatomy or affect the radiotherapy field. A less predictable consideration is the change of dose distribution during MRL treatments. Administration of radiotherapy inside a perpendicular magnetic field will make secondary electrons susceptible to the Lorentz effect, causing redirection and ultimately observation of the electron return effect (ERE), which is more serious for high-field magnets, as used in the Elekta Unity. When secondary electrons are created at air-tissue interfaces, the mobile electrons in the air are susceptible to influence from the magnetic field and will travel in a circular path perpendicular to the field. Dosimetrically, this creates a widening of the penumbra of the beam or asymmetric dose distributions and can increase skin dose [69,85,86]. These effects are typically measured during commissioning of individual machines and accounted for in radiotherapy planning, but must be considered prior to treatment [87]. This ERE can result in unwanted skin effects [88].

Among these practical limitations, the most constricting is treatment time. Multiple feasibility trials have failed prespecified time limitations, typically from a prolonged adaptation process [76,89]. Longer treatment time is not without consequences; restless patients may be tempted to move and eliminate any of the benefit provided from adaptive therapy. Even though treatment times have decreased significantly since these initial experiences [90,91], there remain continued efforts to increase the efficiency of adaptive treatment [92,93,94].

Finally, workflows that utilize MRI for treatment guidance are always limited by inherent flaws in the imaging modality itself. A notable example is the lack of electron density information that is necessary for radiotherapy planning and typically provided by CT. Electron density is paramount to Monte Carlo simulations of dose deposition. For this reason, patients treated on these MRLs typically undergo both CT and MRI simulations, with both sets of scans used for treatment planning for better estimation of dose distribution. Another issue common to MRI is geometric distortion. The source of this warping can be inherent inhomogeneities in the magnetic field, the motion of objects within the field during imaging, or distance from the isocenter [43]. Any systemic distortion is typically measured in quality analysis and accounted for during treatment planning but can remain a confounder to accurate radiotherapy.

Hurdles to the adoption of MRgRT can be encountered within the machine itself or in the implementation of the man-power or human systems that surround it. To coordinate these complicated treatments, the MRgRT program will require cooperation across multiple disciplines and occupations. Patient and operator safety require different protocols when working in a magnetic field as well as MR-compatible equipment in the treatment room. Radiation therapists trained on traditional CT-based workflows will have to undergo education regarding the use and interpretation of MRI [95]. Further, there is significant workforce capital required, mainly composed of additional physician effort and dedicated physicists, which can significantly increase the cost of treatment.

The first step in overcoming them are identifying the limitations. Only after they have been thoroughly analyzed can solutions be created, validated, and implemented.

## 4. Future Applications and Expected Advancements in MRL Technology

Multiple trials have been conducted or have commenced with the purpose of investigating clinical improvements provided by MRI-based workflows and treatments. Trials continue to show safe, well-targeted dosimetry with associated reduction of toxicity and equivalent or improved oncologic outcomes [75,76,89,96,97].

Much of the work to come will be improving the feasibility of treatment, with the goal of reducing treatment time and increasing patient tolerance of MRgRT. The majority of treatment time comes from the segmentation and associated replanning or optimization associated with adaptive treatments [98]. One such avenue for improvement comes from the creation and implementation of auto segmentation and auto-planning algorithms. The former programs can delineate structures after training on a large subset of volumes and have been slowly implanted in standard radiotherapy for some time. Recent implementation of a machine learning model to create auto segmented volumes showed a reduction in contouring time of 93%, as the planning oncologist was required only to edit the volumes [99]. Advances in volume delineation are also being made in the MR realm. One group used longitudinal studies from patients treated on an MRL to deformably apply old contours to successive scans and found progressively improving accuracy of said contours [100].

A step beyond auto-contouring would be auto-planning. In a cohort of 50 patients, an automated treatment planning system generated head and neck radiotherapy plans with a higher percentage of autogenerated plans deemed clinically acceptable than traditionally created plans, albeit on CT based therapy [101]. Similar approaches can reduce the time of contouring and planning and produce increased feasibility of these machines.

As mentioned, a current weakness of MRgRT is the continued need for CT simulation to gather information regarding electron density of tissues near the site of treatment. To overcome this, significant effort has been put into the estimation of Hounsfield Units based on MRI [102]. Typically termed pseudo-CT or synthetic CT, creating an electron density estimation of MRI could avoid the need for two separate simulation scans. The simplest method of doing so is using bulk density override, applying an average electron density over a large field for a crude estimate. As expected, these can lead to inaccurate estimates in more heterogeneous fields [103,104]. Moving one step further is the method of atlas-based production of synthetic CT. An MRI series is matched with a close approximation of a standardized MRI atlas with a known CT correlate, and structures are deformably used to estimate their corresponding CT values [105]. This method has been proven to approximate CT density well, with multiple examples focusing on prostate atlases [105,106]. Finally, voxel-based techniques use multiple sequences of MRI to best approximate CT density on a per-voxel basis [102]. Hybrid methods combining all three techniques have also been described and are useful in managing atypical anatomy [107,108]. Each method has its own limitations and situational benefits.

Overall, image-guided radiotherapy has been the catalyst to evolution within the field of radiation oncology. The greatest endeavor to push the boundaries of the therapeutic index must be the incorporation of MRLs into clinical workflows and improvement of MR-based treatment feasibility. With time, these will be addressed by increases in efficiency and streamlining workflows. The use of MRgRT will continue to require multidisciplinary cooperation and investigation. Safety measures, quality assurance, and patient acceptance will remain the greatest hindrance to the adoption of these machines. Despite such challenges, the MRgRT may hold the key to taking the next leap in treatment for radiotherapy. Clinical outcomes associated with MRL will be watched carefully in coming years.

## Abbreviations

**Abbreviation **	**Definition**
2D	Two-dimensional
3D	Three-dimensional
ATP	Adapt-to-Position
ATS	Adapt-to-Shape
CBCT	Cone-beam computed tomography
cGy	Centigray
CT	Computed Tomography
dB	Decibel
DNA	Deoxyribonucleic acid
ERE	Electron Return Effect
Gy	Gray
IGRT	Image-guided Radiotherapy
IMRT	Intensity-modulated radiation therapy
Linac	Linear Accelerator
MLC	Multileaf Collimator
MR	Magnetic Resonance
MRgRT	Magnetic Resonance-Guided Radiotherapy
MRI	Magnetic Resonance Imaging
MRL	Magnetic Resonance Linear Accelerator
MU	Monitor Unit
MV	Megavolt
MVCT	Megavoltage Computed Tomography
NMR	Nuclear Magnetic Resonance
OAR	Organ at Risk
PET	Positron Emission Tomography
PTV	Planning Target Volume
RF	Radiofrequency
RT	Radiotherapy
S&S	Step-and-Shoot
SNR	Signal-to-Noise Ratio
SPECT	single-photon emission computed tomography
SW	Sliding Window
T	Tesla
T1	Longitudinal relaxation time
T2	Transverse relaxation time
TPS	Treatment planning system
VMAT	Volumetric Modulated Arc Therapy
